# Females of a gift-giving spider do not trade sex for food gifts: a consequence of male deception?

**DOI:** 10.1186/s12862-017-0953-8

**Published:** 2017-05-15

**Authors:** Irene Pandulli-Alonso, Agustín Quaglia, Maria J. Albo

**Affiliations:** 1 Laboratorio de Etología, Ecología y Evolución, Instituto de Investigaciones Biológicas Clemente Estable, Avenida Italia, 3318 Montevideo, Uruguay; 2 Laboratorio de Arbovirus—Instituto de Virología “Dr. J. M. Vanella”–Facultad de Ciencias Médicas, Universidad Nacional de Córdoba, Córdoba, Argentina

**Keywords:** Antagonistic coevolution, Female counteract, Worthless gifts, Polyandry, *Paratrechalea ornata*

## Abstract

**Background:**

Polyandry is commonly maintained by direct benefits in gift-giving species, so females may remate as an adaptive foraging strategy. However, the assumption of a direct benefit fades in mating systems where male gift-giving behaviour has evolved from offering nutritive to worthless (non-nutritive) items. In the spider *Paratrechalea ornata,* 70% of gifts in nature are worthless. We therefore predicted female receptivity to be independent of hunger in this species. We exposed poorly-fed and well-fed females to multiple males offering nutritive gifts and well-fed females to males offering worthless gifts.

**Results:**

Though the treatments strongly affected fecundity, females of all groups had similar number of matings. This confirms that female receptivity is independent of their nutritional state, i.e. polyandry does not prevail as a foraging strategy.

**Conclusions:**

In the spider *Pisaura mirabilis*, in which the majority (62%) of gifts in nature are nutritive, female receptivity depends on hunger. We therefore propose that the dependence of female receptivity on hunger state may have evolved in species with predominantly nutritive gifts but is absent in species with predominantly worthless gifts.

**Electronic supplementary material:**

The online version of this article (doi:10.1186/s12862-017-0953-8) contains supplementary material, which is available to authorized users.

## Background

Evolution and maintenance of polyandry has been extensively studied during the last decades, and so far, costs and benefits have been empirically tested and verified in several species of vertebrates and invertebrates [1–8]. It is known that in polyandrous species selective pressures on males’ sexual traits become more intense the more partners the females mate with and thus the more uncertain paternity is [1]. Females may be interested in access to territories, parental care, food gifts or protection because all these male-provided benefits increase their fecundity and survival, overall enhancing successful reproduction [9–12]. However, due to different adverse factors resulting in limited food availability or low body condition, males may not be able to provide the goods that females prefer. Instead, they may maximize their success by reducing their costs, e.g. by deception [13, 14]. If females are unable to discriminate such deceptive behaviours, polyandry would be maintained in spite of sexual antagonism resulting in reproductive advantages for males and suboptimal mating rates for females [15].

By mating multiple times females from nuptial gift-giving species can gain direct benefits in the form of food gifts, gathering resources that improve their fitness (i.e. fecundity, hatching success, survival) [10, 16–18]. It has been argued that originally nuptial gifts appeared as “paternal investment”, in which nutrients supplied by the gift are used by females to increase the number and/or success of offspring [10, 19, 20]. However, there is also evidence that in some species males can use gifts to manipulate female behaviour [21]. Such is the case in species in which males’ gift-giving behaviour evolved into offering of non-nutritive items, also known as worthless gifts [22–24]. The evolution of worthless gifts and the subsequent selection pressures on females to counteract the deception has rarely been studied [25].

The Neotropical gift-giving spider *Paratrechalea ornata* (Trechaleidae) is exceptional for studying sexual selection in relation to gift content. This is because field data sampled along the reproductive season from three different populations in Uruguay indicate that 70% of nuptial gifts are worthless [26]. Although males can court without a gift, they experience a reduced female acceptance rate, shorter mating duration as well as lower sperm storage in the female spermathecae [26–28]. This creates selective pressure on the males to offer nuptial gifts. Males can provide nutritive gifts by capturing an insect prey and wrapping it in silk [29], but when no prey is available, they may wrap inedible items like plant parts, seeds or prey leftovers, in silk to produce worthless gifts [26]. Females are polyandrous [30] but they can only recognize the gift content after they have grabbed the gift and accepted to mate. Mating duration is extremely short (c. 1 min) in this species, and it has been suggested that females are unable to recognize the gift content in such a short time. Hence, this deceptive behaviour allows males to mate as successfully as males with nutritive gifts, i.e. they obtain similar frequencies of acceptances and similar mating durations [26].

Deception by offering worthless gifts has also been described in the Palaearctic spider *Pisaura mirabilis* with an occurrence of 38% in the field [24]. As most of the gifts in this species are nutritive there is selection on females for remating multiply because it increases their fecundity and the hatching success of their eggs [31]. However, due to mating costs (lowered fecundity and hatching success; reduced hatchling size [31]) females benefit from remating multiply only at suboptimal prey availabilities. With high prey availability the female may meet the nutritional demands at low foraging costs and no mating costs, so that the fecundity benefit for the females of an additional mating is low. This may result in a correlation between female hunger state and receptivity, so that polyandry contributes to the female’s foraging strategy depending negatively on prey availability [31–33]. In the case of *Paratrechalea ornata*, however, with high frequency of worthless gifts there is less/no selection for coupling hunger and receptivity as long as mating costs are non-trivial. Though we do not know the magnitude of mating costs in this species, by comparison with *P. mirabilis* we expected little or no influence of hunger state on female receptivity. We propose the general hypothesis that, all else equal, female receptivity depends on hunger in mating systems with mostly nutritive gifts but not in mating systems with a majority of worthless gifts. Here, we study the situation in *P. ornata* and expect a result that contrasts with those already published for *P. mirabilis* [31–33]. Following this prediction we exposed well-fed females of *P. ornata* to multiple males offering either nutritive or worthless gifts and predicted that females would accept similar number of matings across both gift groups. Thus, females receiving nutritive gifts will acquire more food and most probably achieve higher fitness (i.e. oviposition, fecundity, hatching success) compared to those receiving worthless gifts, but the number of matings should be independent of the nutritional state of the females. If the hypothesis is true, then also females under extremely limited foraging opportunities should not significantly increase the overall number of matings in order to access more food. Thus, we also exposed poorly fed females to multiple males offering nutritive gifts. These females were expected to obtain lower fecundity than the well-fed females, as they would need to use some of the food to compensate for their low condition. Fitness measures of females under limited foraging opportunities will ultimately strengthen our understanding of the reproductive consequences of receiving worthless gifts.

## Methods

We collected juveniles, subadults and adults at night during the reproductive season of 2013 and 2014 from Santa Lucía River, Paso del Molino, Lavalleja, Uruguay (34°16′40.10″ S, 55° 14′00.80″W). Immature individuals were placed in a climate room at 25.02 °C (± 0.11 SE) to accelerate their development. We provided water in a cotton wool daily and twice a week we fed them with fruitflies (*Drosophila* sp.). Once individuals reached adulthood we transferred them to the experimental room at an average temperature of 21.33 °C (± 0.16 SE). Here they were subjected to the same feeding regimen during the next 15 days. With this procedure we ensured that all spiders were sexually mature and the females receptive [34]. After this period, we started experiments where we continued feeding adult males twice a week, while females were maintained in two feeding groups until oviposition: well fed and poorly fed. We fed well-fed females with 10 fruitflies every day, while poorly fed females only ate from gifts offered by males. We verified that as consequence of the food received via matings (1 fly gift per mating) and the daily feeding regimen (10 fruitflies per day) in relation to the number of experiments, the total food events (number of food occurrence: gifts + fruitflies/ number of experiments) by females was different among groups (mean ± SE): Well fed-Nutritive gift (2.59 ± 0.05) > Well fed-Worthless gift (1.97 ± 0.01) > Poorly fed- Nutritive gift (0.58 ± 0.04) (GLM (p): *X* ^2^_2, 63_ = 438.93, *p* < 0.0001). We carried out mating experiments with all groups during both years. We used only unmated females and we also planned to use only unmated males, but due to the unexpectedly high number of matings per female, we would have needed more than 800 males in total, a sample size impossible to reach with this spider species. Therefore, we also used adult males from the field and sometimes males were used more than once. Females and males were randomly assigned to each of the three experimental groups, eliminating any possible effect of individual variation on the between groups comparison. We verified that repetition of males occurred similarly among the three groups, and most females mated equally with different males (90% approx; F_2, 63_ = 2.14, *p* = 0.13).

Multiple matings were obtained by exposing different groups of females to males with different types of gift every two days until eggsac construction. With this procedure, we allowed females to encounter numerous males carrying silk wrapped gifts throughout the reproductive period, and thus they had many mating opportunities before constructing the first eggsac. Following a previous protocol [26, 28], one experimental group was the Well fed-Nutritive gift group (*N* = 21), where well-fed females were exposed to males offering nutritive gifts, which consisted of a recently captured housefly (*Musca domestica*). A second group was the Well fed-Worthless gift group (*N* = 22), where well-fed females were exposed to males offering worthless gifts consisting of the exuviae of a mealworm (*Tenebrio molitor* larva). A third consisted of poorly fed females which had not received fruitflies and could feed only from nutritive fly gifts offered by males (Poorly fed-Nutritive gift, *N* = 21). Initially, we wanted an experimental design, which also included poorly fed females exposed to males offering worthless gifts. However, in preliminary assays these females ended up in very bad body condition and failed to accept matings and construct eggsacs. Hence, due to ethics issues related to animal care we did not carry out experiments with this group. Experimental protocols were carried out in accordance with the general approved guidelines for animal behaviour.

We performed the experiments in transparent plastic jars (15 cm diameter × 9 cm height) in which we simulated natural conditions by covering the bottom with pebbles and water. We placed females in the experimental jars 24 h before experiments, allowing them to habituate and deposit silk that stimulate male courtship and silk wrapping of the gift [35]. Trials followed a fixed procedure: first we enclosed the female inside a glass vial (3 cm diameter and 8 cm height) in the experimental jar. Subsequently, we placed the male in the jar with female silk, allowing him 20 min to detect female sex pheromones and start wrapping the gift material (a live housefly given to the male or exuviae of a mealworm placed in the jar). If the male did not wrap the fly or exuviae we allowed physical contact with the female but not the mating, as we simulated female rejection by pushing her away with a paintbrush. Female rejection stimulates silk wrapping of the gift [27]. We then enclosed the female again and left the male on the silk for another 20 min; if the male still did not start silk wrapping we replaced him with another male. Up to three males were tested at each mating session if necessary. This procedure avoided potential individual incompatibilities and secured that all males offered wrapped gifts. When the male had a wrapped gift we finally released the female and allowed contact between the sexes and mating. We registered all behaviours during 30 min if no mating occurred or until 5 min after the mating was finished.

The behavioural response variables included: frequency of mating, latency of female acceptance, mating duration, sexual cannibalism and gift stealing (i.e. if females grasped the gift and ran away without mating). Latency of acceptance was considered only when mating occurred and was measured from the moment we allowed contact between the sexes and until the female grasped the gift. Mating duration was calculated from the total number of matings of each female and measured as the total duration of all pedipalp insertions, each one lasting from intromission until disengagement. The frequency of sexual cannibalism and gift stealing events was recorded for each female, including several cases by the same female.

The fitness response variables included: latency of oviposition, fecundity and hatching success. We calculated latency of oviposition as the days taken by females to construct the eggsac since the first accepted mating. Once females constructed the eggsac, we placed them under light bulbs (60 W) in order to increase luminosity and temperature (mean ± SE: 28.47 ± 0.14 °C) for three hours at midday. This procedure improves spiderling emergence and has previously been used with other spider species [24, 36]. We provided water and fed females with fruitflies daily. Once spiderlings emerged we counted them and opened the eggsac to also record the unhatched eggs. Fecundity was calculated as the sum of spiderlings and unhatched eggs, and hatching success as the proportion of spiderlings from the total fecundity. If females abandoned the eggsac before the spiderlings emerged, we opened it and counted the number of unhatched eggs inside; in nine cases this was not possible because the females had eaten the eggsac.

### Statistical analyses

We performed statistical analyses using free platform R [37]. Considering our experimental design, we performed Generalized lineal mixed models (GLMM), with response variables being mating and fitness parameters, experimental groups used as fixed effects, female ID as a random effect, and female age as covariate. This considered repeated measures structure within females for fitness parameters controlling for the effect of female ID and age; while also a female effect random structure for the different levels of mating parameters. We explored the distribution of the raw data for each variable to account for the potential error family distribution [38]. We examined using a GLMM (Binomial) frequency of mating, cannibalism, gift stealing and hatching success. Latency of oviposition was analysed using Generalized least square (GLS), while fecundity was analysed using GLMM (Poisson). We performed LMM (LogNormal) to analyse effects on latency of acceptance and mating duration. All the models were validated through the exploration of residual errors with graphical tools [38]. Raw data are presented as Additional file 1.

## Results

### Mating effects

**Fig. 1:**
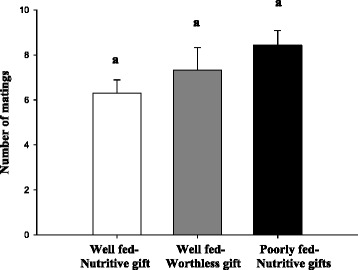
Number of matings from well-fed females receiving fly gifts (Well fed-Nutritive gift, *N* = 21), well-fed females receiving worthless gifts (Well fed-Worthless gift, *N* = 22) and poorly fed females receiving fly gifts (Poorly fed-Nutritive gift, *N* = 21). Data are given as means ± standard error; different letters indicate statistical differences among groups

The frequency of accepted matings was not significantly different among the females of the three experimental groups (Table 1, Fig. 1). Latency of acceptance and mating duration were also similar showing no statistical differences among the groups (Tables 1 and 2).

**Table 1:** GLMM and LMM comparing mating effects among groups (well-fed females receiving fly gifts (Well fed-Nutritive gift), well-fed females receiving worthless gifts (Well fed-Worthless gift) and poorly fed females receiving fly gifts (Poorly fed-Nutritive gift))

MATING EFFECTS	N	Fixed effects	Random effects-Female ID		
		Group	Intercept Std Dev	Age Std Dev	Correlation structure
Frequency of mating	857	*X* ^*2*^_*Wald*_ = 5.25, *p* = 0.06	0.61	0.02	0.10
Latency of acceptance (min)	471	*X* ^*2*^_*Wald*_ = 3.70, *p* = 0.16	0.16	0.01	0.35
Mating duration (min)	471	*X* ^*2*^_*Wald*_ = 4.97, *p* = 0.08	0.53	0.02	0.31
Cannibalism (yes/no)	856	*X* ^*2*^_*Wald*_ = 6.75, *p* = **0.03**	1.43	0.06	0.01
Gift stealing	855	*X* ^*2*^_*Wald*_ = 15.58, *p* < **0.001**	1.05	0.06	−0.01

In all models, Group was considered as fixed effects, female ID (*N* = 64) as random effects and female age as covariate. Significant *p*-values are shown in bold

**Table 2:** Latency of mating acceptance (averaged per female), mating duration (summed per female) and cannibalism occurrence among well-fed females receiving fly gifts (Well fed-Nutritive gift), well-fed females receiving worthless gifts (Well fed-Worthless gift) and poorly fed females receiving fly gifts (Poorly fed-Nutritive gift)

	Well fed-Nutritive gift	Well fed-Worthless gift	Poorly fed- Fly gift
Latency of acceptance (min)	11.70 ± 1.41 a (*n* = 21)	8.11 ± 1.04 a (*n* = 22)	5.21 ± 0.55 a (*n* = 21)
Mating duration (min)	11.39 ± 2.66 a (*n* = 21)	7.56 ± 1.38 a (*n* = 22)	11.52 ± 1.35 a (*n* = 21)
Cannibalism (yes/no)	8/225 a	10/330 a	22/302 b

Data are given as mean ± standard error; different letters indicate statistical differences

We observed (but did not quantify) that poorly fed females were less active, i.e. walking and contacting males less often during courtship and mating than well-fed females.

**Fig. 2:**
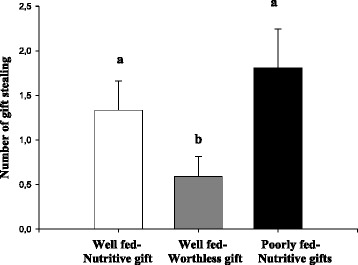
Number of gift stealing performed by females during courtship from well-fed females receiving fly gifts (Well fed-Nutritive gift, *N* = 21), well-fed females receiving worthless gifts (Well fed-Worthless gift, *N* = 22) and poorly fed females receiving fly gifts (Poorly fed-Nutritive gift, *N* = 21). Data are given as mean ± standard error; different letters indicate statistical differences among groups

Few females (4% overall) attacked and cannibalized males in the three groups; poorly fed females cannibalized more males than females in the other groups (Table 1; Table 2). Some females stole the gift from courting males, meaning that they accepted the gift but ran away without mating. The occurrence of gift stealing showed differences among groups as follows: Poorly fed-Nutritive gift = Well fed-Nutritive gift > Well fed-Worthless gift (Table 1; Fig. 2).

### Fitness effects

**Fig. 3:**
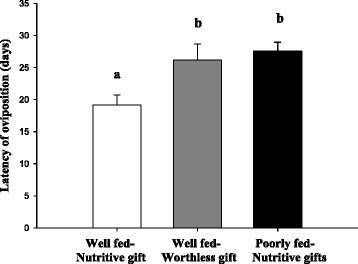
Latency of oviposition from well-fed females receiving fly gifts (Well fed-Nutritive gift, *N* = 21), well-fed females receiving worthless gifts (Well fed-Worthless gift, *N* = 22) and poorly fed females receiving fly gifts (Poorly fed-Nutritive gift, *N* = 21). Data are given as mean ± standard error; different letters indicate statistical differences among groups

**Fig. 4:**
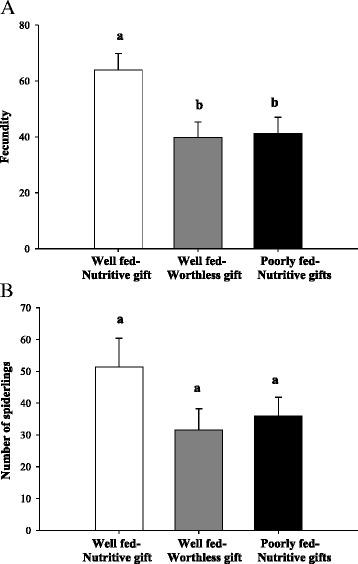
**a** Fecundity (hatched eggs + unhatched eggs) and **b** Number of spiderlings (hatched eggs), from well-fed females receiving fly gifts (Well fed-Nutritive gift, N _Fecundity_ = 18 and N _No. spiderlings_ = 17), well-fed females receiving worthless gifts (Well fed-Worthless gift, N _Fecundity_ = 19 and N _No. spiderlings_ = 19) and poorly fed females receiving fly gifts (Poorly fed-Nutritive gift, N _Fecundity_ = 18 and N _No. spiderlings_ = 17 respectively). Data are given as mean ± standard error; different letters indicate statistical differences among groups

Twenty-one well-fed females receiving fly gifts, 22 well-fed females receiving worthless gifts and 21 poorly fed females receiving fly gifts constructed an eggsac. Latency of oviposition was statistically different among the three groups (Table 3). Well-fed females receiving fly gifts oviposited earlier than females in the other groups (Fig. 3). Also, well-fed females receiving fly gifts had elevated fecundity (spiderlings + unhatched eggs) compared to females in the other groups (Table 3; Fig. 4a). Number of spiderlings was larger in the group of well-fed females receiving fly gifts compared to the other two groups, but with no statistical differences (Table 3; Fig. 4b). Hatching success (spiderlings / fecundity) did not differ among groups (Table 3).

**Table 3:** GLMM comparing fitness effects among groups (well-fed females receiving fly gifts (Well fed-Nutritive gift), well-fed females receiving worthless gifts (Well fed-Worthless gift) and poorly fed females receiving fly gifts (Poorly fed-Nutritive gift))

FITNESS EFFECTS	N	Fixed effects	Random effects-Female ID
		Group	Intercept Std Dev
Latency of oviposition (days)	64	*X* ^*2*^_*Wald*_ = 11.45, *p* = **0.003**	-
Fecundity	55	*X* ^*2*^_*Wald*_ = 10.43, *p* = **0.005**	0.56
No. spiderlings	53	*X* ^*2*^_*Wald*_ = 0.92, *p* = 0.63	1.62
Hatching success	53	*X* ^*2*^_*Wald*_ = 0.03, *p* = 0.98	11.33

In all models, Group was considered as fixed effects and female ID (*N* = 64) as random effects. Significant *p*-values are shown in bold

## Discussion

It is known that females from nuptial gift-giving species often mate with multiple males, trading sex for food gifts and consequently gaining fitness benefits [16]. They can even modulate their mating rate based on what is optimal under certain ecological conditions [16, 31, 39]. For instance, empirical examples have also shown that when food is scarce, polyandrous females can drastically increase the number of matings [31, 39, 40], and even compete for potential mates that offer food resources in the form of gifts [41–44]. But, in mating systems with high frequency of deception by worthless gifts, like in the spider *P. ornata*, little or no influence of hunger state on female receptivity may be predicted. Our results showed that all female groups had similar mating rate irrespective of feeding condition, and even poorly fed females did not significantly increase the number of matings with males offering nutritive gifts. Thus, our results support the hypothesis of behavioural differences between mating systems that differ in the relative frequency of nutritive and worthless gifts. In the gift-giving spider *P. mirabilis*, worthless gifts occur at low frequency (38%) in the field [24] and females in low nutritional condition double their mating rate compare to those in high condition [31–33]. In such a mating system, females gain food from multiple matings/gifts, resulting in an increase of their fitness [31]. In the case of *P. ornata*, we did not find support for the hypothesis of polyandry as an adaptive foraging strategy. We do not have solid information to understand how mating behaviour has changed over evolutionary time in this species, but one possibility is that control of female receptivity may have changed due to evolution of male deception. As both *P. ornata* and *P. mirabilis* are polyandrous it seems possible that polyandry is maintained by other reasons than direct benefits. Alternatively, it can be argued that the lack of association between female hunger state and receptivity may be mediated by low food availability in the population, which also leads to high frequency of worthless gifts. In this scenario, high female mating activity may have been favoured under food restricted conditions, so that the more they mate, the higher the chances of consuming at least a few nutritive gifts. This requires, however, that mating costs in *P. ornata* are much lower than in *P. mirabilis*. The much shorter mating duration (c. 1 min against c. 70 min) make this a realistic possibility. Nevertheless, we would need further studies evaluating costs of mating in this species, as well as food availably and body condition in nature.

Polyandry may be maintained by indirect benefits interacting with direct benefits [45, 46]. If in *P. ornata* the nuptial gift is a reliable signal of positive male attributes, then females may gain genetic benefits from accepting males offering gifts even if these are worthless. For instance, beyond gift content, gift-giving males usually invest in silk wrapping which involves costs such as time and energy, and those in poor body condition are usually limited in this behaviour [36, 47]. Thus, silk wrapping represents an honest indicator of some male attributes and quality, and females would benefit from mating with “good wrappers” [48, 49].

Due to the females’ inability to recognize the gift content before accepting the mating [26], there is a high risk of being deceived by courting males. It would be advantageous for females to recognize the gift content before mating, as by accepting only males with nutritive gifts females can significantly increase their reproductive outcome. We found that those receiving the highest nutritional benefits oviposited earlier and obtained higher fecundity than the others. By constructing the first eggsac early these females will probably have more eggsacs and therefore, more offspring along the reproductive season. Effects of food gifts on oviposition have been suggested before in this and another gift-giving spider [31, 45], however, these studies were unable to disentangle the effects of food and sperm. Here, we can discard an effect of sperm as all groups had similar mating number and mating duration, whereas differences in food intake lead to differences in the latency of oviposition. Thus, the variation in female fitness parameters (oviposition and fecundity) must be explained mainly by the variation in the amount food consumed. Effects of from nuptial gifts on fecundity and hatching success are well-known in some insects [10, 16]. When females receive nutritive gifts their net food intake increases and they can use it in their own metabolism as well as in egg production [50, 51]. Preliminary results verify that the food acquired from the nuptial gifts can be distributed into somatic and reproductive tissues in *P. ornata*, mostly incorporated into eggs and silk of the eggsac (Costa-Schmidt unpublished data).

Indeed, in our experiments females receiving worthless gifts experienced a reduction in fitness like females limited in their foraging opportunities. Under this scenario, they would need to balance the potential costs of mating under limited food supply. Then, one potential tactic that females can perform to compensate these costs and increase their food consumption is to reject the mating and cannibalize the male [52, 53]. Although cannibalism occurred in low proportion in all groups, we found that poorly fed females cannibalized males more frequently than females from the other groups. Another possible female tactic to avoid mating costs is to steal gifts from males during courtship. We found that poorly fed females stole the gifts more often compared to the well-fed group receiving worthless gifts. However, well-fed females receiving nutritive gifts also presented high percentage of gifts stealing (similar to poorly fed females) suggesting that it may be a matter of gift type. This is not necessarily an indication that females recognize the gift content during courtship, because it can also be a consequence of the silk wrapping of the gift. It has been suggested that the silk helps males to better grasp and keep control on the gift [54, 55]; further experiments are needed to understand whether males invest differently in silk depending on gift type.

## Conclusions

This study is the first to discuss whether male deception may influence female receptivity and mating rate in gift-giving species. In the particular case of the spider *P. ornata* females can still acquire fitness advantages from nutritive gifts. However, we conclude that polyandry does not seem to prevail as a foraging strategy in *P. ornata*, and suggest that the dependence of female receptivity on hunger in gift-giving species depends on the level of male deception. To verify this, future studies using less extreme feeding conditions would need to focus on whether females can (before mating) evaluate and respond differentially to nutritive vs. worthless gifts according to their nutritional state.

## Additional file

Additional file 1: Raw data. (PDF 197 kb)
