# Supplementary material for: Females of a gift-giving spider do not trade sex for food gifts: a consequence of male deception?
Source: BMC Evol Biol. 2017 May 15;17:112. doi: 10.1186/s12862-017-0953-8 (PMC5433081; doi:10.1186/s12862-017-0953-8)
Supplement: Additional file 1: — Raw data. (PDF 197 kb) [file 12862_2017_953_MOESM1_ESM.pdf]

MATING EFFECTS

Group	Number of experiments	Number of accepted matings	Proportion of different males	Total food occurrence
Well fed-Nutritive gift	7	3	1,00	2,29
Well fed-Nutritive gift	8	7	1,00	2,88
Well fed-Nutritive gift	9	7	0,86	2,78
Well fed-Nutritive gift	7	2	0,50	2,43
Well fed-Nutritive gift	8	6	0,83	2,75
Well fed-Nutritive gift	11	7	0,71	2,55
Well fed-Nutritive gift	10	3	1,00	2,30
Well fed-Nutritive gift	10	3	1,00	2,20
Well fed-Nutritive gift	14	12	0,83	2,79
Well fed-Nutritive gift	16	10	0,60	2,75
Well fed-Nutritive gift	11	5	0,80	2,64
Well fed-Nutritive gift	16	3	1,00	2,19
Well fed-Nutritive gift	11	11	0,73	3,00
Well fed-Nutritive gift	16	7	1,00	2,44
Well fed-Nutritive gift	10	8	0,75	2,80
Well fed-Nutritive gift	15	8	1,00	2,53
Well fed-Nutritive gift	12	5	1,00	2,42
Well fed-Nutritive gift	7	4	1,00	2,57
Well fed-Nutritive gift	9	6	1,00	2,67
Well fed-Nutritive gift	8	7	1,00	2,75
Well fed-Nutritive gift	10	8	1,00	2,70
Well fed-Worthless gift	9	1	1,00	1,89
Well fed-Worthless gift	12	8	1,00	2,00
Well fed-Worthless gift	10	8	0,88	1,90
Well fed-Worthless gift	13	6	1,00	2,00
Well fed-Worthless gift	13	7	0,71	2,00
Well fed-Worthless gift	12	9	0,78	2,00
Well fed-Worthless gift	27	21	0,38	1,96
Well fed-Worthless gift	21	15	0,53	2,00
Well fed-Worthless gift	26	17	0,41	1,96
Well fed-Worthless gift	19	6	1,00	1,95
Well fed-Worthless gift	11	3	0,67	2,00
Well fed-Worthless gift	24	9	0,89	2,00
Well fed-Worthless gift	16	7	1,00	2,00
Well fed-Worthless gift	15	4	1,00	2,00
Well fed-Worthless gift	19	6	1,00	1,95
Well fed-Worthless gift	18	6	1,00	2,00
Well fed-Worthless gift	13	4	1,00	1,85
Well fed-Worthless gift	8	6	1,00	1,88
Well fed-Worthless gift	10	6	1,00	1,90
Well fed-Worthless gift	16	4	1,00	2,00
Well fed-Worthless gift	9	3	1,00	2,00
Well fed-Worthless gift	9	5	1,00	2,00

MATING EFFECTS

Group	Number of experiments	Number of accepted matings	Proportion of different males	Total food occurrence
Poorly fed-Nutritive gift	9	5	0,80	0,56
Poorly fed-Nutritive gift	11	6	1,00	0,55
Poorly fed-Nutritive gift	12	3	0,67	0,25
Poorly fed-Nutritive gift	18	7	0,86	0,39
Poorly fed-Nutritive gift	12	5	0,80	0,42
Poorly fed-Nutritive gift	17	11	0,91	0,65
Poorly fed-Nutritive gift	16	10	0,70	0,63
Poorly fed-Nutritive gift	12	6	0,83	0,50
Poorly fed-Nutritive gift	16	8	0,75	0,50
Poorly fed-Nutritive gift	18	11	0,82	0,61
Poorly fed-Nutritive gift	17	6	1,00	0,35
Poorly fed-Nutritive gift	15	10	1,00	0,67
Poorly fed-Nutritive gift	12	9	1,00	0,75
Poorly fed-Nutritive gift	11	9	1,00	0,82
Poorly fed-Nutritive gift	13	12	1,00	0,92
Poorly fed-Nutritive gift	14	9	0,89	0,64
Poorly fed-Nutritive gift	13	11	0,91	0,85
Poorly fed-Nutritive gift	15	8	1,00	0,53
Poorly fed-Nutritive gift	18	10	0,82	0,56
Poorly fed-Nutritive gift	11	5	1,00	0,45
Poorly fed-Nutritive gift	22	16	0,88	0,73

MATING EFFECTS

Group	Mating duration (min)	Latency of acceptance (min)	Cannibalism	Gift stealing
Well fed-Nutritive gift	9,6	23,03	0	2
Well fed-Nutritive gift	16	8,47	0	1
Well fed-Nutritive gift	18,1	9,51	0	0
Well fed-Nutritive gift	2,1	0,35	3	2
Well fed-Nutritive gift	5,4	19,58	0	2
Well fed-Nutritive gift	18,8	5,94	1	0
Well fed-Nutritive gift	4,9	6,23	0	2
Well fed-Nutritive gift	1,4	13,07	0	6
Well fed-Nutritive gift	57,8	4,63	0	0
Well fed-Nutritive gift	11,9	15,41	0	2
Well fed-Nutritive gift	4,5	3,78	0	3
Well fed-Nutritive gift	8,7	13,23	0	0
Well fed-Nutritive gift	22,2	10,98	0	0
Well fed-Nutritive gift	7,3	8,71	2	2
Well fed-Nutritive gift	9,5	16,31	0	1
Well fed-Nutritive gift	2,2	10,15	1	3
Well fed-Nutritive gift	0,8	27,26	1	1
Well fed-Nutritive gift	12,7	13,55	0	0
Well fed-Nutritive gift	12,8	8,97	0	0
Well fed-Nutritive gift	6,7	10,60	0	0
Well fed-Nutritive gift	5,8	15,96	0	1
Well fed-Worthless gift	4,1	6,80	0	0
Well fed-Worthless gift	18,5	4,45	0	0
Well fed-Worthless gift	20,6	10,46	0	0
Well fed-Worthless gift	7	15,33	1	0
Well fed-Worthless gift	4	5,43	0	0
Well fed-Worthless gift	12,1	16,50	0	0
Well fed-Worthless gift	23,2	6,99	1	0
Well fed-Worthless gift	12,3	6,55	2	0
Well fed-Worthless gift	12,1	5,06	0	4
Well fed-Worthless gift	4,4	6,22	2	2
Well fed-Worthless gift	3,4	5,67	0	0
Well fed-Worthless gift	3,6	7,93	0	2
Well fed-Worthless gift	5,8	1,90	3	1
Well fed-Worthless gift	1,9	6,98	0	0
Well fed-Worthless gift	2,4	12,58	0	2
Well fed-Worthless gift	1,4	7,50	1	0
Well fed-Worthless gift	3	23,00	0	1
Well fed-Worthless gift	11,7	3,72	0	0
Well fed-Worthless gift	7,1	6,25	0	0
Well fed-Worthless gift	1,1	3,73	0	1
Well fed-Worthless gift	4,3	6,70	0	0
Well fed-Worthless gift	2,3	8,72	0	0

MATING EFFECTS

Group	Mating duration (min)	Latency of acceptance (min)	Cannibalism	Gift stealing
Poorly fed-Nutritive gift	4,3	4,23	2	1
Poorly fed-Nutritive gift	10,2	3,75	0	2
Poorly fed-Nutritive gift	16,8	1,30	1	7
Poorly fed-Nutritive gift	9	2,93	0	5
Poorly fed-Nutritive gift	7,3	5,23	0	4
Poorly fed-Nutritive gift	13,6	4,56	1	1
Poorly fed-Nutritive gift	15,4	3,92	2	4
Poorly fed-Nutritive gift	5,8	2,67	1	1
Poorly fed-Nutritive gift	17,2	6,59	2	1
Poorly fed-Nutritive gift	9,8	6,27	2	2
Poorly fed-Nutritive gift	4,9	5,08	0	5
Poorly fed-Nutritive gift	9,6	4,94	1	1
Poorly fed-Nutritive gift	15,6	5,83	2	0
Poorly fed-Nutritive gift	12,2	4,36	0	0
Poorly fed-Nutritive gift	31	6,68	0	0
Poorly fed-Nutritive gift	12,1	9,79	1	0
Poorly fed-Nutritive gift	9,7	13,04	1	1
Poorly fed-Nutritive gift	9,1	3,67	2	1
Poorly fed-Nutritive gift	7,3	4,16	3	0
Poorly fed-Nutritive gift	3,4	2,85	1	1
Poorly fed-Nutritive gift	17,8	7,59	0	1

FITNESS EFFECTS

Group	Latency of oviposition (days)	Fecundity	Number of spiderlings	Number of Unhatched eggs
Well fed-Nutritive gift	13	75	75	0
Well fed-Nutritive gift	16	100	100	0
Well fed-Nutritive gift	18	52	39	13
Well fed-Nutritive gift	15	118	118	0
Well fed-Nutritive gift	14			
Well fed-Nutritive gift	19	72	0	72
Well fed-Nutritive gift	8			
Well fed-Nutritive gift	7			
Well fed-Nutritive gift	27	39		39
Well fed-Nutritive gift	34	59	59	0
Well fed-Nutritive gift	24	55	55	0
Well fed-Nutritive gift	24	15	0	15
Well fed-Nutritive gift	22	65	65	0
Well fed-Nutritive gift	28	71	1	70
Well fed-Nutritive gift	18	50	0	50
Well fed-Nutritive gift	30	25	22	3
Well fed-Nutritive gift	22	52	50	2
Well fed-Nutritive gift	14	67	59	8
Well fed-Nutritive gift	18	62	57	5
Well fed-Nutritive gift	15	96	96	0
Well fed-Nutritive gift	17	78	78	0
Well fed-Worthless gift	11	57	57	0
Well fed-Worthless gift	18	35	35	0
Well fed-Worthless gift	17			
Well fed-Worthless gift	18	55	55	0
Well fed-Worthless gift	26			
Well fed-Worthless gift	22	8	0	8
Well fed-Worthless gift	53			
Well fed-Worthless gift	36	25	25	0
Well fed-Worthless gift	51	35	35	0
Well fed-Worthless gift	35	64	62	2
Well fed-Worthless gift	14	23	23	0
Well fed-Worthless gift	40	19	19	0
Well fed-Worthless gift	28	48	33	15
Well fed-Worthless gift	28	21	0	21
Well fed-Worthless gift	35	38	38	0
Well fed-Worthless gift	30	30	0	30
Well fed-Worthless gift	20	23	0	23
Well fed-Worthless gift	15	79	79	0
Well fed-Worthless gift	19	104	104	0
Well fed-Worthless gift	26	37	12	25
Well fed-Worthless gift	16	45	15	30
Well fed-Worthless gift	18	10	8	2

FITNESS EFFECTS

Group	Latency of oviposition (days)	Fecundity	Number of spiderlings	Number of Unhatched eggs
Poorly fed-Nutritive gift	18	88	88	0
Poorly fed-Nutritive gift	22	38	38	0
Poorly fed-Nutritive gift	21	17		17
Poorly fed-Nutritive gift	37	20	0	20
Poorly fed-Nutritive gift	24	7	1	6
Poorly fed-Nutritive gift	30			
Poorly fed-Nutritive gift	26	18	13	5
Poorly fed-Nutritive gift	24			
Poorly fed-Nutritive gift	28	71	71	0
Poorly fed-Nutritive gift	36	82	35	47
Poorly fed-Nutritive gift	27			
Poorly fed-Nutritive gift	30	49	48	1
Poorly fed-Nutritive gift	24	46	31	15
Poorly fed-Nutritive gift	22	32	31	1
Poorly fed-Nutritive gift	26	17	17	0
Poorly fed-Nutritive gift	27	51	51	0
Poorly fed-Nutritive gift	26	45	45	0
Poorly fed-Nutritive gift	30	53	52	1
Poorly fed-Nutritive gift	35	65	53	12
Poorly fed-Nutritive gift	22	34	34	0
Poorly fed-Nutritive gift	44	10	3	7
